# Optimal cut-off values for anthropometric measures of obesity in screening for cardiometabolic disorders in adults

**DOI:** 10.1038/s41598-020-68265-y

**Published:** 2020-07-09

**Authors:** Pawel Macek, Malgorzata Biskup, Malgorzata Terek-Derszniak, Halina Krol, Jolanta Smok-Kalwat, Stanislaw Gozdz, Marek Zak

**Affiliations:** 1 Institute of Health Sciences, Collegium Medicum, Jan Kochanowski University, 25-317 Kielce, Poland; 2 Department of Epidemiology and Cancer Control, Holycross Cancer Centre, 25-734 Kielce, Poland; 3 Department of Rehabilitation, Holycross Cancer Centre, 25-734 Kielce, Poland; 4 Clinical Oncology Clinic, Holycross Cancer Centre, 25-734 Kielce, Poland; 5 Research and Education Department, Holycross Cancer Centre, 25-734 Kielce, Poland

**Keywords:** Health care, Public health, Population screening

## Abstract

Excessive accumulation of body fat (BF) promotes obesity, whilst posing a significant health hazard. There being no agreed, optimal quantifying methods, application of BF variable in clinical practice is not deemed an effective assessment option. The study, involving 4,735 patients (33.6% men), aged 45–64, aimed to identify optimal cut-off values for anthropometric indicators of obesity to evaluate cardiometabolic risk. A minimum *P*-value approach was applied to calculate the cut-offs for BF%. Threshold values for body mass index (BMI), waist circumference (WC), waist-to-hip ratio (WHR), and waist-to-height (WHTR) ratio, facilitating optimal differentiation of cardiometabolic risk, were based on BF%, expressed as a binary classifier. The newly estimated cut-off values for predicting cardiometabolic risk, based on BMI, were lower than the referential obesity thresholds, whereas the threshold values of WC, WHR, and WHTR were higher. Apart from dyslipidemia, the odds of cardiometabolic disorders were higher, when the anthropometric indicators under study exceeded the cut-off points in both sexes. The proposed cut-offs proved instrumental in predicting cardiometabolic risk, whilst highlighting diagnostic and clinical potential of BF%, whereas BMI boasted the highest predictive potential. Cardiometabolic risk also proved significantly higher even in the overweight patients.

## Introduction

According to the World Health Organization (WHO), obesity entails excessive and life-threatening accumulation of body fat (BF), which poses a significant risk factor for cardiometabolic disorders^1–5^. Common anthropometric indicators of obesity are grounded in the assessment of body fat (BF)^6^. Current BMI-based cut-off values for obesity were established against the data mined from prospective trials involving Caucasian participants, in which „hard outcome” was represented by mortality^7,8^. Although the application of BMI in assessing obesity is practical, its true value remains dubious^9,10^. It is well-worth noting that BMI does not actually address physiological differences in BF related to sex, age, race-ethnicity, and should not be applied in patients aged 14 and younger^11–15^. A far better solution would therefore consist in carrying out a direct or indirect measurement of BF, even though proposing a reliable classification of obesity based on such a measurement would still leave the major problem unresolved^16^. Despite BMI correlating with BF, it feels rather challenging to establish a BF cut-off threshold based on the cut-off values for BMI^17–19^. Some investigators propose a similar solution, though, frequently based on the obesity-related comorbidities, rather than a single BMI evaluation^20–22^. Regretfully, some of the authors do get caught in the same erroneous paradigm regarding the cut-off values for BF, attributing them to WHO recommendationns^23^.

Since obesity, especially abdominal obesity, is associated with cardio-metabolic disorders, epidemiological studies make use of central obesity indicators such as waist circumference (WC), waist-to-hip ratio (WHR), and waist-to-height ratio (WHTR), apart from to BMI^24,25^. Both WHO and American Heart Association recommend the use of WC in screening for cardiometabolic risk^26^. Some inaccuracies are reported, however, as to the best anatomical site for waist-to-hip measurement. Furthermore, the cut-off points tend to vary, depending on the subjects' gender and ethnicity, respectively. Other studies indicate WHTR to be a better predictor of cardio-metabolic risk than BMI in the individuals suffering from diabetes, metabolic syndrome, and other cardiovascular risk factors (CRFs)^27,28^. According to Ashwell et al., making use of WHTR facilitates the identification of early health risks, especially in the individuals affected by central obesity. It also proves a valuable indicator in the detection of excessive BF accumulation in adolescents with normal BMI, in menopausal women, and in various screening studies, especially those pursued in populations characterised by high risk of central obesity and cardiovascular diseases (CVDs). Hence, compliance with the "keep your waist circumference to less than half your height" principle is clearly postulated^29,30^. Since body height is relatively stable in adults, WHTR changes are assumed to reflect the changes in WC, while WHR is more sensitive to any changes in the waist and hip regions. Despite the ongoing controversy, WHO recommends making use of BMI for an initial obesity assessment, whereas the actual distribution of BF is postulated to be assessed on the basis of WC or WHR values^31^.

Although BF can be measured through numerous techniques steadily gaining in popularity, most of the studies on obesity are based on the indirect measurements; this being attributable to a more universal appeal of BMI^32–36^. Regretfully in large cohort studies, the data on body height and weight needed to have BMI calculated, are acquired through a direct, or a telephone survey^37–39^, rendering them dubious at best. Admittedly, BF measurement is also prone to measurement errors^40^, which seem to be more acceptable when obesity is defined in line with the actual BF content, rather than BMI alone.

Direct comparison of BMI and BF is non-feasible in clinical terms, which poses yet another appreciable challenge for physicians^41,42^. To the best of our knowledge, the proposed cut-off points for BF for women are higher than the ones for men, whereas BMI cut-off values recommended by WHO remain equal for both sexes^26,43–45^. Consequently, assuming that BF is associated with metabolic dysregulation regardless of body mass, some of the patients, especially women, may be misclassified on the basis of against their BMI, and consequently become exposed to an increased absolute risk of CVDs in the future^46–48^. At the same time, uncertainty associated with selecting the most accurate means of adiposity assessment may discourage both BF measurement and its regular monitoring in routine clinical practice^49^.

The study aimed to identify of optimal cut-off values for anthropometric indicators of obesity to assess cardiometabolic risk in adults.

## Results

Mean values for body mass, BMI, WC, WHR, WHTR, SBP, DBP, FBG, TG, as well as the frequency of hypertension, diabetes mellitus, and ≥ 2 CRFs, overweight and obesity, and alcohol consumption was significantly higher in men. Mean values of BF%, as well as HDL-C, LDL-C, and TC concentration were significantly higher in women. The underlying data of single and clustered CRFs, stratified by gender, regarding different anthropometric indicators, with standard cut-offs for obesity, are comprised in Supplementary Table S1.

In both sexes, the mean values for anthropometric indicators, as well as SBP, DBP, FBG and TG were higher when BF% exceeded the threshold (Table 1). However, in both sexes, the mean values of HDL-C were higher in the individuals with BF% below the set threshold. Differences between the mean values of LDL-C and TC, depending on cut off values for BF%, were insignificant. The frequency of single and clustered CRFs was significantly higher in men and women participants, with BF% exceeding the cut-off point. Conversely, among the behavioural factors under study, the frequency of smoking and MVPA was higher, either in men or women with BF% under the threshold values. In the case of BF above the cut-off points, the frequency of alcohol consumption was similar in both sexes.

**Table 1:** Basic characteristics of the study group, stratified by gender and optimal BF% cut-off values.

	Men		*P*^1^	Women		*P*^2^
	BF < 25.8%	BF ≥ 25.8%		BF < 37.1%	BF ≥ 37.1%	
	(n = 782)	(n = 808)		(n = 1,801)	(n = 1,344)	
Age (years)	54.3 ± 5.5	55.7 ± 5.5	3.5E−07	54.2 ± 5.4	56.5 ± 4.9	2.2E−16
Height (cm)	174.4 ± 6.4	173.3 ± 6.3	0.0016	159.7 ± 5.8	160.2 ± 5.8	0.0127
Weight (kg)	78.3 ± 9.3	92.6 ± 11.5	2.2E−16	63.1 ± 7.3	79.9 ± 10.5	2.2E−16
BMI (kg/m^2^)	25.7 ± 2.4	30.8 ± 3.3	2.2E−16	24.8 ± 2.8	31.2 ± 4.1	2.2E−16
WC (cm)	92.0 ± 7.0	104.8 ± 8.9	2.2E−16	80 ± 7.6	95.2 ± 9.3	2.2E−16
WHR	0.9 ± 0.1	1.0 ± 0.1	2.2E−16	0.8 ± 0.1	0.9 ± 0.1	2.2E−16
WHTR	0.5 ± 0.1	0.6 ± 0.1	2.2E−16	0.5 ± 0.1	0.6 ± 0.1	2.2E−16
BF (%)	21.9 ± 3.0	31.3 ± 4.7	2.2E−16	31.2 ± 4.6	41.4 ± 3.2	2.2E−16
SBP (mm/Hg)	139.4 ± 17.8	145.4 ± 18.2	4.0E−11	131.4 ± 18.1	139.1 ± 18.9	2.2E−16
DBP (mm/Hg)	82.4 ± 9.3	86.1 ± 10.7	1.7E−13	78.5 ± 9.7	82.0 ± 9.9	2.2E−16
FBG (mg/dl)	97.2 ± 15.1	105.4 ± 22.6	2.2E−16	92.4 ± 14.3	99.4 ± 19.8	2.2E−16
HDL-C (mg/dl)	54.3 ± 12.7	50.3 ± 11.7	4.1E−11	65.7 ± 15.3	58.3 ± 13.2	2.2E−16
LDL-C (mg/dl)	126.2 ± 33.3	124.7 ± 33.8	0.3543	128.1 ± 33.3	129.0 ± 33.7	0.4673
TC (mg/dl)	203.1 ± 37.2	203.3 ± 39.0	0.9107	213.8 ± 36.7	212.1 ± 38.0	0.2141
TG (mg/dl)	112.6 ± 56.4	141.9 ± 69.3	2.2E−16	99.5 ± 50.1	124.2 ± 54.8	2.2E−16
Hypertension, n (%)	296 (37.9)	507 (62.8)	3.2E−23	561 (31.2)	784 (58.3)	1.8E−52
Dyslipidemia, n (%)	593 (75.8)	651 (80.6)	0.0221	1,445 (80.2)	1,080 (80.4)	0.9311
Diabetes mellitus, n (%)	43 (5.5)	123 (15.2)	2.3E−10	57 (3.2)	119 (8.9)	6.6E−12
≥ 1 risk factor, n (%)	677 (86.6)	767 (94.9)	8.1E−09	1562 (86.7)	1,256 (93.5)	9.9E−10
≥ 2 risk factors, n (%)	237 (30.3)	446 (55.2)	1.2E−23	472 (26.2)	660 (49.1)	5.5E−40
BMI ≥ 25, n (%)	500 (63.9)	798 (98.8)	7.0E−72	808 (44.9)	1,317 (98.0)	1.4E−217
BMI ≥ 30, n (%)	27 (3.5)	434 (53.7)	4.7E−108	57 (3.2)	744 (55.4)	3.5E−242
Smoker, n (%)	171 (21.9)	125 (15.5)	0.0011	360 (20.0)	177 (13.2)	5.1E−07
Drinker, n (%)	710 (90.8)	735 (91.0)	0.9049	1535 (85.2)	1,124 (83.6)	0.2823
MVPA, n (%)	301 (38.5)	233 (28.8)	4.6E−05	661 (36.7)	382 (28.4)	1.1E−06

Data are presented as mean ± standard deviation, unless otherwise indicated.

*BMI* body mass index, *WC* waist circumference, *WHR* waist-to-hip ratio, *WHTR* waist- to-height ratio, *BF* body fat, *SBP* systolic blood pressure, *DBP* diastolic blood pressure, *FBG* fasting blood glucose, *TC*, total cholesterol, *TG* triglyceride, *LDL-C* low-density lipoprotein, *HDL-C* high-density lipoprotein, *MVPA* moderate to vigorous physical activity in leisure.

*P*^1^ and *P*^2^, statistically significant differences between adopted BF% groups for men and women respectively.

Cardiometabolic risk based on BF% was noted in 90% of the participants classified as obese, in line with BMI. When obesity was diagnosed with the application of WC, WHR, and WHTR, cardiometabolic risk ranged from 40 to 70%. Regretfully, more than 30% of men, and 25% of women without obesity, as established against their BMI, turned out to be at risk of cardiometabolic disorders, when based on their BF%. As opposed to BMI assessment, in 88–96% of respondents, who were not diagnosed as obese, there was no cardiometabolic risk indeed, as established against the WC, WHR, and WHTR obesity indicators (Table 2).

**Table 2:** BF% in non-obesity and obesity categories based on BMI, WC, WHR, WHTR, stratified by gender.

	Men		Women	
	BF < 25.8%	BF ≥ 25.8%	BF < 37.1%	BF ≥ 37.1%
BMI < 30, n (row%)	755 (66.9)	374 (33.1)	1744 (74.4)	600 (25.6)
BMI ≥ 30, n (row%)	27 (5.9)	434 (94.1)	57 (7.1)	744 (92.9)
WC normal, n (row%)	451 (89.7)	52 (10.3)	853 (96.3)	33 (3.7)
WC obesity, n (row%)	331 (30.5)	756 (69.6)	948 (42.0)	1,311 (58.0)
WHR normal, n (row%)	49 (89.1)	6 (10.9)	203 (88.3)	27 (11.7)
WHR obesity, n (row%)	733 (47.8)	802 (52.3)	1598 (54.8)	1,317 (45.2)
WHTR normal, n (row%)	176 (96.2)	7 (3.8)	885 (93.4)	63 (6.7)
WHTR obesity, n (row%)	606 (43.1)	801 (56.9)	916 (41.7)	1,281 (58.3)

*BMI* body mass index (kg/m^2^), *WC* waist circumference (cm), *WHR* waist-to-hip ratio, *WHTR* waist- to-height ratio, *BF* body fat (%).

**Figure 1:**
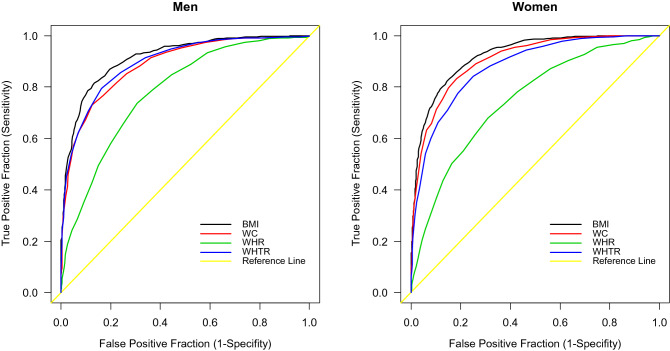
ROC curves for BMI, WC, WHR, and WHTR in screening for CRFs in men and women. *BMI* body mass index, *WC* waist circumference, *WHR* waist-to-hip ratio, *WHTR* waist- to-height ratio.

Estimated gender-specific cut-offs values for BF% were used as binary classifiers for establishing the new cut-off points for the anthropometric indicators under study regarding obesity (Table 3). In both sexes, optimal cut-offs for BMI were lower than the values indicating obesity. However, the cut-off points for other anthropometric indicators were higher than common threshold values for obesity. At the same time, BMI was noted to have the highest sensitivity and specificity for at least one CRF (Fig. 1). The probability of 1 CRF was six times higher in men, and over five times higher in women, when BMI exceeded the established cut-off values. The lowest probability of 1 CRF was noted in the case of WHR. These probabilities increased twofold in both men and women with WHR above the threshold. The differences between AUCs of all indicators under study were statistically significant at α = 0.01.

**Table 3:** Cut-off values for anthropometric indicators of obesity, based on optimal BF% cut-offs for screening CRFs, separately for men and women.

	AUC (95% CI)	Optimal cut-off	Sensitivity (%)	Specificity (%)	Youden (%)	DLR (+)	DLR (−)	*P*
**Men**								
BMI	0.915 (0.902, 0.929)	28.1	81.6	86.6	68.1	6.1	0.2	0.000001
WC	0.887 (0.871, 0.903)	100.0	73.0	87.3	60.4	5.8	0.3	0.000001
WHR	0.781 (0.759, 0.803)	0.96	73.8	69.4	43.2	2.4	0.4	0.000001
WHTR	0.895 (0.880, 0.910)	0.57	79.5	83.6	63.1	4.9	0.2	0.000001
**Women**								
BMI	0.922 (0.913, 0.931)	27.5	83.3	84.5	67.8	5.4	0.2	0.000001
WC	0.905 (0.895, 0.915)	87.0	83.3	81.6	64.9	4.5	0.2	0.000001
WHR	0.747 (0.730, 0.764)	0.85	67.9	69.0	36.9	2.2	0.5	0.000001
WHTR	0.878 (0.866, 0.889)	0.54	84.1	75.1	59.1	3.4	0.2	0.000001

*BMI* body mass index, *WC* waist circumference, *WHR* waist-to-hip ratio, *WHTR* waist- to-height-ratio, *AUC* area under the curve, *DLR* (+) positive diagnostic likelihood ratio, *DLR* (−) negative diagnostic likelihood ratio.

Based on the newly estimated cut-offs for anthropometric indicators, some changes were noted (as compared to the standard cut-offs) in the allocation of respective cases into groups affected and non-affected by CRFs risk, assessed against individual BF% (Table 4). Some improvement was noted with regard to specificity of BMI, and sensitivity of WC, WHR, and WHTR. Depending on the anthropometric index, cardiometabolic risk was observed in 71–86% of men, and in 63–80% of women, whose obesity index values were above the thresholds estimated in the present study.

**Table 4:** BF% in newly estimated non-obesity and obesity categories based on BMI, WC, WHR, WHTR, stratified by gender.

	Men			Women	
	BF < 25.8%	BF ≥ 25.8%		BF < 37.1%	BF ≥ 37.1%
BMI < 28.1	677 (82.0)	149 (18.0)	BMI < 27.5	1522 (87.1)	225 (12.9)
BMI ≥ 28.1	105 (13.7)	659 (86.3)	BMI ≥ 27.5	279 (20.0)	1,119 (80.0)
WC < 100.0	683 (75.8)	218 (24.2)	WC < 87.0	1,470 (86.7)	225 (13.3)
WC ≥ 100.0	99 (14.4)	590 (85.6)	WC ≥ 87.0	331 (22.8)	1,119 (77.2)
WHR < 0.96	543 (71.9)	212 (28.1)	WHR < 0.85	1,287 (73.3)	470 (26.8)
WHR ≥ 0.96	239 (28.6)	596 (71.4)	WHR ≥ 0.85	514 (37.0)	874 (63.0)
WHTR < 0.57	654 (79.8)	166 (20.2)	WHTR < 0.54	1,352 (86.3)	214 (13.7)
WHTR ≥ 0.57	128 (16.6)	642 (83.4)	WHTR ≥ 0.54	449 (28.4)	1,130 (71.6)

*BMI* body mass index (kg/m^2^), *WC* waist circumference (cm), *WHR* waist-to-hip ratio, *WHTR* waist-to-height ratio, *BF* body fat (%).

The odds for single and clustered CRFs were strongly associated with new cut-off values for anthropometric indicators. The underlying data of single and clustered CRFs, stratified by different anthropometric indicators with newly estimated gender-specific cut-offs in screening for cardiometabolic disorders, are comprised in Supplementary Table S2. In both men and women, the prevalence pattern of single and clustered CRFs, based on the estimated cut-offs for the anthropometric indicators under study, compared to the standard thresholds, was similar. Based on the standard cut-offs for BMI, the prevalence of cardiometabolic disorders was higher in both non-obese and obese subjects, compared to the estimated BF% cut-offs for this indicator. Conversely, for the remaining analysed anthropometric indicators (WC, WHR, WHTR), the frequency of CRFs, in line with the cut-offs estimated in this study was higher than in line with commonly used cut-offs for the anthropometric indicators under study (Supplementary Table S3).

**Figure 2:**
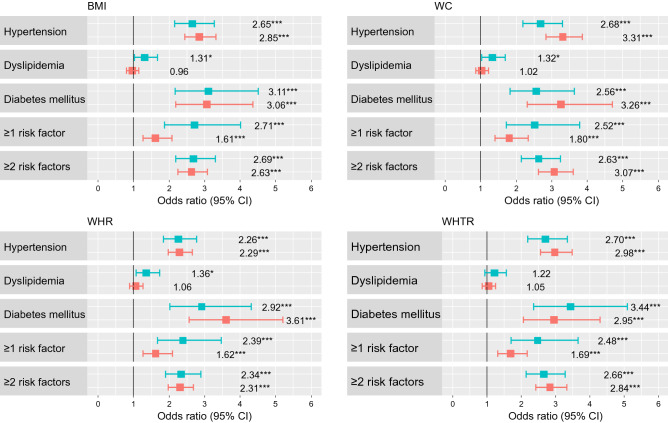
Adjusted ORs (95% CIs) of CRFs vs. non-CRFs associated with anthropometric indicators for men (green) and women (red). Notes: Adjustment comprises: age; smoking status categorised as non-smoker (never smoker and former smoker) or smoker (current smoker); drinking status categorised as (never drinker and former drinker) or drinker (current drinker); moderate to vigorous physical activity in leisure categorised as yes or no. *BMI* body mass index, *WC* waist circumference, *WHR* waist-to-hip ratio, *WHTR* waist-to-height ratio. *P < 0.05, **P < 0.01, ***P < 0.001.

Based on the unadjusted (Supplementary Table S4), and adjusted regression models (Fig. 2), with the exception of dyslipidemia, the odds for CRFs were significantly higher in both sexes, when the anthropometric indicators under study exceed the cut off threshold comprised in Table 3. This was clearly manifest in the case of diabetes mellitus. Regardless of gender, depending on anthropometric indicators, the odds for diabetes mellitus were 2.5–3.5 times higher in men and women with BMI, WC, WHR, or WHTR over the cut-off values. The odds for ≥ 1 CRFs were higher in men than in women, regardless of the anthropometric indicator under study.

The odds for CRFs based on the standard thresholds of obesity indicators were similar to those estimated on the basis of the newly estimated cut-offs (Table 5). Due attention was drawn, however, to the higher values of standard errors, which affected the 95% CIs. In particular, the assessment of the odds for diabetes mellitus based on WHR in both genders, and WHTR in men, seemed downright useless.

**Table 5:** Adjusted ORs (95% CIs) of CVD risk factors vs. non-CVD risk factors associated with various anthropometric indicators of obesity, stratified by gender.

Men	BMI < 30.0	BMI ≥ 30.0	WC < 94.0	WC ≥ 94.0	WHR < 0.85	WHR ≥ 0.85	WHTR < 0.50	WHTR ≥ 0.50
Hypertension	1.00 (ref)	2.84 (2.25, 3.59)***	1.00 (ref)	2.85 (2.28, 3.59)***	1.00 (ref)	3.96 (2.09, 8.18)***	1.00 (ref)	3.67 (2.55, 5.38)***
Dyslipidemia	1.00 (ref)	1.56 (1.18, 2.08)**	1.00 (ref)	1.24 (0.95, 1.60)	1.00 (ref)	1.06 (0.53, 2.00)	1.00 (ref)	1.54 (1.05, 2.22)*
Diabetes mellitus	1.00 (ref)	2.95 (2.12, 4.12)***	1.00 (ref)	3.55 (2.22, 6.00)***	1.00 (ref)	5.37 (1.16, 95.59)	1.00 (ref)	10.03 (3.13, 61.21)**
≥ 1 risk factor	1.00 (ref)	3.38 (2.07, 5.89)***	1.00 (ref)	2.08 (1.46, 2.95)***	1.00 (ref)	1.34 (0.54, 2.86)	1.00 (ref)	2.86 (1.84, 4.39)***
≥ 2 risk factors	1.00 (ref)	2.88 (2.30, 3.62)***	1.00 (ref)	2.92 (2.31, 3.70)***	1.00 (ref)	5.09 (2.43, 12.42)***	1.00 (ref)	3.91 (2.64, 5.97)***

Women	BMI < 30.0	BMI ≥ 30.0	WC < 80.0 cm	WC ≥ 80.0 cm	WHR < 0.75	WHR ≥ 0.75	WHTR < 0.50	WHTR ≥ 0.50
Hypertension	1.00 (ref)	3.28 (2.75, 3.90)***	1.00 (ref)	2.93 (2.45, 3.52)***	1.00 (ref)	2.82 (2.03, 4.01)***	1.00 (ref)	2.83 (2.37, 3.39)***
Dyslipidemia	1.00 (ref)	1.08 (0.88, 1.34)	1.00 (ref)	1.03 (0.84, 1.25)	1.00 (ref)	1.19 (0.85, 1.63)	1.00 (ref)	1.05 (0.86, 1.29)
Diabetes mellitus	1.00 (ref)	3.68 (2.68, 5.07)***	1.00 (ref)	3.26 (2.01, 5.64)***	1.00 (ref)	5.88 (1.85, 35.77)*	1.00 (ref)	2.88 (1.82, 4.81)***
≥ 1 risk factor	1.00 (ref)	2.15 (1.55, 3.05)***	1.00 (ref)	1.61 (1.26, 2.05)***	1.00 (ref)	1.77 (1.22, 2.53)**	1.00 (ref)	1.57 (1.23, 2.01)***
≥ 2 risk factors	1.00 (ref)	3.02 (2.55, 3.59)***	1.00 (ref)	2.73 (2.26, 3.31)***	1.00 (ref)	2.69 (1.89, 3.93)***	1.00 (ref)	2.74 (2.28, 3.32)***

Adjustment comprises: age; smoking status categorised as non-smoker (never smoker and former smoker) or smoker (current smoker); drinking status categorised as (never drinker and former drinker) or drinker (current drinker); moderate to vigorous physical activity in leisure categorised as yes or no.

*BMI* body mass index (kg/m^2^), *WC* waist circumference (cm), *WHR* waist-to-hip ratio, *WHTR* waist-to-height ratio.

*P < 0.05; **P < 0.01; ***P < 0.001.

## Discussion

The present study aimed to establish optimal cut-off values for anthropometric indicators of obesity, with a view to screening for cardiometabolic risk. Approx. 30% of men and 25% of women from high cardiometabolic risk group, based on BF%, were not classified as obese, following BMI assessment only. Conversely, more than 90% of either women or men with high cardiometabolic risk, based on BF%, were classified as obese, in line with BMI assessment. Some 40–70% study participants from high cardiometabolic risk group (BF%) were classified as obese, in line with WC, WHR, and WHTR assessment. As opposed to BMI, cardiometabolic risk was not appreciably elevated in 90% of both sexes, i.e. individuals not diagnosed with obesity based on the above-referenced criteria. In both sexes, specificity and sensitivity for screening at least one CRF were the highest for BMI, and the lowest for WHR. In both sexes, when the indicators under study exceeded the threshold values, the odds for cardiometabolic disorders were higher.

In clinical terms, diagnosis of obesity within its own right is useless, as it is not capable of discriminating against potentially adverse health consequences^41^. Interestingly, regardless of sex and ethnicity, similar indicators of overall adiposity boast comparable discriminative value^39^. Similarly, regardless of any confounding factors, excessive accumulation of adipose tissue triggers similar health consequences^42^. Cut-off values for BF% or adjusted cut-off values for a classic anthropometric indicator of overall adiposity are varied, depending on the methodology actually applied, population sample under study, gender, age, or race-ethnicity^18,19^. Once a certain threshold is exceeded, though, morbidity patterns seem to remain unaffected. It follows that proposals to have the threshold values for BMI or other anthropometric indicators adjusted, may not sufficiently be justified^20,43,44^.

Even though we are not disputing the obvious sex differences regarding the anthropometric indicators, individual gains in body mass or body circumference alone do not pose an actual health hazard. As health hazard is triggered by increased BF, an increase in body mass caused by overall adiposity, or increase in waist circumference caused by central adiposity, are generally acknowledged as health-threatening^26,45^. Nevertheless, physiological differences in body habitus depending on sex, age, or race-ethnicity stand for a separate issue^46^. Application of obesity as an exclusive cut-off value for novel, race-ethnicity-specific threshold values for BMI would bring little benefit. Apart from individual differences in obesity, BMI is affected by other bodily features (i.e. musco-skeletal mass), status. It is therefore postulated that other indicators of the phenotype be applied with significant factors in terms of individual health a view to addressing more effectively bodily proportions, both on an individual and population level^11^.

Anthropometric indicators, including BMI, WC, WHR, or WHTR, are crucial for cardiometabolic risk assessment, albeit some authors argue that their discriminative value depends on age, sex, study cohort and race-ethnicity^47,48^. All of the variables under study are crude indicators of obesity, assessed in an indirect manner^49^. The choice of the most accurate indicator, best suited for assessing cardiometabolic risk still remains a point of contention. Some investigators regard BMI as the most accurate indicator of overall adiposity, whereas others claim that WC and WHR, which represent the extent and distribution of adiposity, are most suitable^50–52^. Regardless of the type of obesity, in most of the studies the discriminative value of screening for cardiometabolic risk is associated with the accuracy of adiposity assessment, even an indirect one^9^.

The International Atherosclerosis Society (IAS) and the International Chair on Cardiometabolic Risk (ICCR) have brought together a body of evidence indicating that assessment of the obesity-related cardiometabolic risk based exclusively on BMI is insufficient^53^. According to IAS and ICCR recommendations, classification of obesity based on both WC and BMI should become a routine diagnostic procedure in clinical practice. According to Ardern et.al.^54^, assessment of cardiometabolic risk based on a single, sex-specific threshold for WC in all BMI categories is insufficient. The WC thresholds, as proposed by the authors within the existing BMI categories, were verified with regard to CVDs risk against the Framingham Risk Score. The brand-new, sex-specific WC thresholds, as put forward in the current study, are identical in men, and closely similar in women, as those recommended by Ardern et al. within the BMI category corresponding to overweight. Considering that the newly proposed sex-specific cut-offs for BMI also indicate a significant rise in cardiometabolic risk in the overweight individuals, the assessment of health outcomes, as recommended by Ross et al., based on BMI and WC as an essential "vital sign", appears of particular consequence^53^.

All in all, an indicator boasting good discriminative ability translates into high diagnostic potential. The downside is, however, that none of the adiposity indicators strictly reflects the actual adiposity. It follows, that discriminative ability of these indicators may appreciably be enhanced by making pertinent adjustments to the cut-off values, in line with BF% based on cardiometabolic risk, with no potential impairment to the diagnostic process at the same time. Failure to have crude obesity indicators corrected by BF% may well result either in underestimation or overestimation of cardiometabolic risk, as screened for on their basis.

There is a perceptible scarcity of reliable test results combining other than the BMI-related indicators of body composition with long-term health outcomes. Since BMI has limited value in differentiating between the lean body mass and adipose tissue, some researchers and clinicians have for years favoured the assertion that purportedly overweight or obesity may paradoxically offer extra protective effects with regard to cardiometabolic disorders^55^. The ensuing confusion might most likely be due to an increased susceptibility of BMI to early disease conditions^34^. It has been proven that diseases in their subclinical stage promote muscle mass loss which is then bound to increase overall percentage of adipose tissue within the body mass, even when it otherwise happens to remain on a stable level^56^. It follows that without taking into account the concomitant diseases, the causal link between low BMI and the incidence of CVDs is a potentially misleading health message.

On the other hand, unavailability of clinical assessment of BF% in the persons with normal BMI may result in ignoring the already existing health hazard in the persons presenting no clinical signs of a disease. In the present study, the BMI cut-offs meant to be applied for discriminating against the individuals affected by at least 1CRF, to be subsequently allocated into respective groups, were established as 28.1 kg/m^2^ and 27.5 kg/m^2^ for men and women, respectively. According to a standard quantification of BMI, both estimated thresholds indicate overweight. The small difference between a commonly acknowledged upper threshold for normal BMI values, and the proposed cut-offs for an increased risk of cardiometabolic disorders (≈ 3 kg/m^2^ men and ≈ 2.5 kg/m^2^ women) may potentially be disregarded, especially in the case of women. An opportunity to have BMI, a commonly acknowledged indicator of obesity, adjusted by the BF value, i.e. the one actually defining obesity, appears a particularly tempting option, especially in clinical terms. The previously referenced availability of BF diagnostic equipment is yet another effectively motivating factor.

Two different approaches are readily distinguishable in the studies addressing the health hazards associated with BF. Some researchers focus on the association of BF with anthropometric indicators of obesity, primarily BMI. Others aim to assess cardiometabolic risk based on B^7,9,49^. Furthermore, a large proportion of investigators, predominantly from Asia, highlight the need to have BMI thresholds verified in the manner taking due account of different population specifics, effectively putting into question its clinical pertinence^44,57^. Clearly, it all originates in the lack of generally endorsed indicator for diagnosing obesity effectively, as well as for screening for cardiometabolic risk with high accuracy. As BF happens to meet both criteria as such an indicator, it well deserves the top recommendation^20,40,42^.

Both the methods applied and the results yielded by our study are meant to appreciably enhance the diagnostic process, with a view to screening for cardiometabolic risk effectively, based on the key indicators of obesity. We do acknowledge, however, that the threshold values established in our study are still subject to be affected by the comorbidity mechanisms specific to the cohort under study (i.e. white Caucasian patients, aged 45–64), effectively precluding straightforward extrapolation into other populations.

In the present study, the best discriminatory capacity for the CRFs risk group was established for BMI. The results actually yielded may well be regarded as posing a certain controversy in obesity research, as recent years have born witness to making use of central obesity indicators being postulated as appreciably better screening tools for individuals at risk of CRFs. Part of the underlying reason for the ensuing controversy may be related to the age of the cohort under study. Persons aged ≥ 60 years accounted for ≈ 25% of the group, whereas those < 50 years of age ≈ 17%. Biological aging carries at least two health risks. Firstly, it is an obesity non-related increase in CRFs risk, and secondly, age-related changes in body composition consisting in an undesirably biased ratio of lean body mass to BF, consequently promoting sarcopenic obesity. It follows that the differences in the discriminating ability of the fat indices under study may actually pertain to the age-related body composition changes. Due to an unavailability of pertinent information, the investigators were unable to take into account any dietary changes in the individuals affected by e.g. diabetes mellitus or hypertension, as recommended by the attending physicians. A potential change in dietary habits may also (apart from age) have resulted in a lower CRFs risk assessment based on central obesity ratio, as compared to BMI. Hence, it may not be ruled out that in older adults BMI is a better suited indicator to be applied in CRFs risk assessment, in comparison with other obesity indicators.

## Conclusions

Optimal cut-offs for anthropometric indicators of obesity, as addressed in some depth in this paper, are appreciably instrumental in screening for cardiometabolic risk, effectively highlighting discriminative power and diagnostic potential of BF%. BMI, a most common obesity indicator, proved to have the highest discriminative ability in both genders. Cardiometabolic risk, calculated in line with BMI, is higher even in the overweight persons, thus warranting application of extra effective preventive and therapeutic measures. Overall, miscalculation of individual health risk has an appreciably detrimental effect upon clinical practice at large. Easy-to-apply anthropometric indicators, structured to aid effective assessment of cardiometabolic risk, boast appreciable potential in streamlining early medical interventions in the public healthcare sector.

## Research design and methods

### The Polish–Norwegian Study (PONS)

The study made use of the source data from the PONS Project, i.e. "Establishment of infrastructure for population health research in Poland". The PONS Project was designed as a cross-sectional study. In the period spanning September 2010–December 2011, pertinent population data were collected, with a view to assessing the key health determinants, and generally addressing the causes of morbidity and mortality in Poland. The study protocol entailed general health assessment (Health Status Questionnaire and physical exam), anthropometric measurements, as well as blood and urine sampling. More detailed information on recruitment for the PONS study is provided elsewhere^58,59^.

### Data verification

The PONS study made use of the data of 4,799 (33.7% men) survey participants, permanent residents of Kielce. Data verification, based on their completeness, prompted the authors to delete all cases of missing data (n = 64) from the database, to ensure effective defining of all established endpoints (Supplementary Figure S1). Ultimately, 4,735 participants (33.6% men) aged 45–64; mean age 55.1 years were recruited as fully eligible to attend the study protocol.

### Anthropometric measurements

Body mass and BF% were measured by S.C.-220 MA body analyzer (accuracy up to 0.1 kg). Body height and circumference were acquired with Seca height measure and a measuring tape (accuracy up to 0.1 cm). WC was acquired at the height of the umbilicus or natural waistline. The hip circumference was measured at the widest part of the hips. BMI was calculated as a quotient of body mass in kilograms and squared height in meters (kg/m^2^). WHR was calculated as the quotient of waist and hip circumference, whereas to calculate WHTR waist circumference was divided by height. Gender-specific standard cut-off values for anthropometric indicators of obesity are shown in Supplementary Table S5. Systolic blood pressure (SBP) and diastolic blood pressure (DBP) were measured with Omron monitor (Model M3 Intellisense) and are presented as the mean of two consecutive measurements.

### Laboratory measurements

Fasting blood glucose (FBG), total cholesterol (TC), high-density lipoproteins (HDL-C), and triglycerides (TG) concentrations were determined in the laboratory against pertinent reference standards, with the aid of enzymatic methods. Low-density lipoprotein cholesterol (LDL-C) level was estimated using Friedewald’s equation for TG level less than 400 mg/dl. Laboratory tests were carried out with CB 350i Wiener Lab (Supplementary Table S6).

### Definitions of the outcomes

SBP ≥ 140 or DBP ≥ 90 mm/Hg or self-reported treatment of hypertension was regarded as hypertension. Diabetes mellitus was diagnosed in the case of FBG > 126 mg/dl or self-reported treatment of diabetes mellitus. TC ≥ 190 mg/dl and/or HDL-C < 40 mg/dl for men (HDL-C < 45 mg/dl for women) and/or LDL-C ≥ 115 mg/dl and/or TG ≥ 150 mg/dl or self-reported dyslipidemia treatment were marked as dyslipidemia. Clustered CRF’s ≥ 1, and ≥ 2, were defined as at least one, or two risk factors, respectively.

### The individual Health Status Questionnaire

International Physical Activity Questionnaire (long version) was used to evaluate moderate to vigorous physical activity in leisure (MVPA). MVPA was estimated based on the number of days and duration of physical activity in leisure time. Smoking status and alcohol consumption were divided into two broad categories, i.e. never (never or former) and current smoker or drinker.

### Statistical analysis

Basic statistics are presented as means ± standard deviations or numbers and proportion, depending on the type of variable under study. The homogeneity of variance was examined by the *F* test. Statistically significant differences between adopted BF% groups, separately in men and women, were estimated with independent *t* test, or Chi-square test. Cut-off values for BF%, which enabled optimal differentiation of cardiometabolic risk, were calculated while making use of the minimum *P*-value approach. It was executed on the basis of series of chi-square independence tests, evaluated for contingency tables created every-time for the pair of variables, i.e. ≥ 1 CRF (at least one of CRF) and dichotomized BF%.

The above-referenced dichotomizations were made for every consecutive unique value of a given sample (i.e. BF% within a sex group), while disregarding the four smallest, and the four greatest unique values, originating in some computational errors. Consequently, the cut-offs for BF% equalled 25.8% for men, and 37.1% for women (Supplementary Figure S2). The estimated threshold values for BF differentiating cardiometabolic risk were used as binary classifiers to establish the cut-off values for BMI, WC, WHR, and WHTR, with the aid of Receiver Operating Characteristics Curve Analyses. In order to assess the cardiometabolic risk based on the select anthropometric indicators, we applied the area under the curve (AUC) ranging between 0 and 1 (a worthless and a perfect test, respectively). Using the DeLong method with Bonferroni correction, pairwise comparison of AUC was pursued, and *P* values < 0.01 were considered statistically significant. The positive likelihood ratio LR (+) and the negative likelihood ratio LR (−) were also determined. ROC curves served to evaluate the discriminatory power of anthropometric indicators to identify the obese patients with at least 1 CRF (hypertension, dyslipidemia, diabetes mellitus). Optimal cut-offs values were established as the point on the curve with maximum Youden’s index (defined as sensitivity + specificity – 1).

Unadjusted and adjusted odds ratios (ORs) and 95% confidence intervals (95% CIs) for CRFs vs. non-CRFs associated with anthropometric measures were calculated with the aid of logistic regression models. Covariates for adjusted ORs were as follows: age, smoking status, alcohol drinking, and MVPA status. Confidence intervals were based on the log-likelihood ratio. *P* values < 0.05 were considered to be statistically significant. Statistical significance is indicated on the graphs with asterisks (*P < 0.05; **P < 0.01; ***P < 0.001). All statistical analyses were performed in R (version 3.5.3).

### Sensitivity analysis

Due to potential changes in the subjects' body composition resulting from specific types of cancer, clinical stage, and the actual method of oncological treatment applied, all cases (n = 191) with confirmed cancer in medical history were excluded from the study database (Supplementary Figure S1). Subsequently, much as in the main analysis, the gender-specific cut-offs for BF% were established with respect to at least one CRF. Consequently, BF% cut-offs for men changed slightly (25.6% as per the sensitivity analysis, and 25.8% as per the main analysis), while for women they remained unaltered (Supplementary Figure S3). No changes were noted in the cut-off values for anthropometric measures of obesity affected by the altered BF% cut-offs in men, based on the sensitivity analysis. Some of the other ROC analysis characteristics (i.e. sensitivity, specificity) (Supplementary Table S7) changed slightly. Therefore, all the analyses at issue were based on the gender-specific cut-offs, estimated against the results yielded by the main analysis.

## Supplementary information

Supplementary Information.
