# Supplementary material for: Optimal cut-off values for anthropometric measures of obesity in screening for cardiometabolic disorders in adults
Source: Sci Rep. 2020 Jul 9;10:11253. doi: 10.1038/s41598-020-68265-y (PMC7347554; doi:10.1038/s41598-020-68265-y)
Supplement: Supplementary file 1 — Supplementary Information. [file 41598_2020_68265_MOESM1_ESM.pdf]

Supplementary materials

Optimal Cut-Off Values for Anthropometric Measures of Obesity in Screening for Cardiometabolic Disorders in Adults

Pawel Macek, Malgorzata Biskup, Malgorzata Terek-Derszniak, Halina Krol, Jolanta Smok-Kalwat, Stanislaw Gozdz, and Marek Zak

Table S1 The underlying data of single and clustered CRFs stratified by gender in the different anthropometric measures with standard cut-offs for obesity

Gender	Variable	Cut-offs	No. in group	Hypertension			Dyslipidemia			Diabetes mellitus			≥1 CRF			≥2 CRFs		
				no	yes	%	no	yes	%	no	yes	%	no	yes	%	no	yes	%
Men	BMI	<30.0	1129	642	487	43.1	266	863	76.4	1050	79	7.0	129	1000	88.6	729	400	35.4
		≥30.0	461	145	316	68.5	80	381	82.6	374	87	18.9	17	444	96.3	178	283	61.4
	WC	<94.0	503	341	162	32.2	116	387	76.9	484	19	3.8	69	434	86.3	374	129	25.6
		≥94.0	1087	446	641	59.0	230	857	78.8	940	147	13.5	77	1010	92.9	533	554	51.0
	WHR	<0.85	55	44	11	20.0	12	43	78.2	54	1	1.8	7	48	87.3	48	7	12.7
		≥0.85	1535	743	792	51.6	334	1201	78.2	1370	165	10.7	139	1396	90.9	859	676	44.0
	WHTR	<0.50	183	143	40	21.9	46	137	74.9	181	2	1.1	35	148	80.9	152	31	16.9
		≥0.50	1407	644	763	54.2	300	1107	78.7	1243	164	11.7	111	1296	92.1	755	652	46.3
Women	BMI	<30.0	2344	1529	815	34.8	468	1876	80.0	2266	78	3.3	283	2061	87.9	1671	673	28.7
		≥30.0	801	271	530	66.2	152	649	81.0	703	98	12.2	44	757	94.5	342	459	57.3
	WC	<80.0	886	682	204	23.0	177	709	80.0	869	17	1.9	131	755	85.2	716	170	19.2
		≥80.0	2259	1118	1141	50.5	443	1816	80.4	2100	159	7.0	196	2063	91.3	1297	962	42.6
	WHR	<0.75	230	185	45	19.6	52	178	77.4	228	2	0.9	42	188	81.7	193	37	16.1
		≥0.75	2915	1615	1300	44.6	568	2347	80.5	2741	174	6.0	285	2630	90.2	1820	1095	37.6
	WHTR	<0.50	948	726	222	23.4	192	756	79.7	928	20	2.1	139	809	85.3	767	181	19.1
		≥0.50	2197	1074	1123	51.1	428	1769	80.5	2041	156	7.1	188	2009	91.4	1246	951	43.3

Abbreviations: BMI, body mass index (kg/m^{-2}); WC, waist circumference (cm); WHR, waist-to-hip ratio; WHTR, waist-to-height ratio.

Table S2 The underlying data of single and clustered CRFs stratified by different anthropometric measures with newly estimated gender-specific cut-offs in predicting cardiometabolic disorders

Gender	Variable	Cut-offs	No. in group	Hypertension			Dyslipidemia			Diabetes mellitus			≥1 CRF			≥2 CRFs		
				no	yes	%	no	yes	%	no	yes	%	no	yes	%	no	yes	%
Men	BMI	<28.1	826	507	319	38.6	192	634	76.8	781	45	5.4	106	720	87.2	567	259	31.4
		≥28.1	764	280	484	63.4	154	610	79.8	643	121	15.8	40	724	94.8	340	424	55.5
	WC	<100.0	901	543	358	39.7	209	692	76.8	844	57	6.3	110	791	87.8	607	294	32.6
		≥100.0	689	244	445	64.6	137	552	80.1	580	109	15.8	36	653	94.8	300	389	56.5
	WHR	<0.96	820	509	311	37.9	183	637	77.7	781	39	4.8	103	717	87.4	564	256	31.2
		≥0.96	770	278	492	63.9	163	607	78.8	643	127	16.5	43	727	94.4	343	427	55.5
	WHTR	<0.57	755	459	296	39.2	178	577	76.4	716	39	5.2	97	658	87.2	515	240	31.8
		≥0.57	835	328	507	60.7	168	667	79.9	708	127	15.2	49	786	94.1	392	443	53.1
Women	BMI	<27.5	1747	1216	531	30.4	339	1408	80.6	1698	49	2.8	222	1525	87.3	1307	440	25.2
		≥27.5	1398	584	814	58.2	281	1117	79.9	1271	127	9.1	105	1293	92.5	706	692	49.5
	WC	<87.0	1695	1216	479	28.3	336	1359	80.2	1651	44	2.6	225	1470	86.7	1302	393	23.2
		≥87.0	1450	584	866	59.7	284	1166	80.4	1318	132	9.1	102	1348	93.0	711	739	51.0
	WHR	<0.85	1566	1129	437	27.9	313	1253	80.0	1525	41	2.6	210	1356	86.6	1208	358	22.9
		≥0.85	1579	671	908	57.5	307	1272	80.6	1444	135	8.5	117	1462	92.6	805	774	49.0
	WHTR	<0.54	1757	1183	574	32.7	354	1403	79.9	1713	44	2.5	224	1533	87.3	1291	466	26.5
		≥0.54	1388	617	771	55.5	266	1122	80.8	1256	132	9.5	103	1285	92.6	722	666	48.0

Abbreviations: BMI, body mass index (kg/m^{-2}); WC, waist circumference (cm); WHR, waist-to hip-ratio; WHTR, waist-to-height ratio.

Table S3 Differences between percentage of cases with CRFs based on standard cut-offs values for obesity anthropometric measures and estimated cut-offs values in predicting cardiometabolic disorders

Gender	Variable	Cut-offs		Hypertension	Dyslipidemia	Diabetes mellitus	≥1 CRF	≥2 CRFs
		Standard	Estimated	Difference (%)	Difference (%)	Difference (%)	Difference (%)	Difference (%)
Men	BMI	<30.0	<28.1	4.5	-0.3	1.5	1.4	4.1
		≥30.0	≥28.1	5.2	2.8	3.0	1.5	5.9
	WC	<94.0	<100.0	-7.5	0.1	-2.5	-1.5	-7.0
		≥94.0	≥100.0	-5.6	-1.3	-2.3	-1.9	-5.5
	WHR	<0.85	<0.96	-17.9	0.5	-2.9	-0.2	-18.5
		≥0.85	≥0.96	-12.3	-0.6	-5.7	-3.5	-11.4
	WHTR	<0.50	<0.57	-17.3	-1.6	-4.1	-6.3	-14.8
		≥0.50	≥0.57	-6.5	-1.2	-3.6	-2.0	-6.7
Women	BMI	<30.0	<27.5	4.4	-0.6	0.5	0.6	3.5
		≥30.0	≥27.5	7.9	1.1	3.2	2.0	7.8
	WC	<80.0	<87.0	-5.2	-0.2	-0.7	-1.5	-4.0
		≥80.0	≥87.0	-9.2	0.0	-2.1	-1.6	-8.4
	WHR	<0.75	<0.85	-8.3	-2.6	-1.7	-4.9	-6.8
		≥0.75	≥0.85	-12.9	0.0	-2.6	-2.4	-11.5
	WHTR	<0.50	<0.54	-9.3	-0.1	-0.4	-1.9	-7.4
		≥0.50	≥0.54	-4.4	-0.3	-2.4	-1.1	-4.7

Abbreviations: BMI, body mass index (kg/m^{-2}); WC, waist circumference (cm); WHR, waist-to-hip ratio; WHTR, waist-to-height ratio.

Table S4 Unadjusted ORs (95% CIs) of CVD risk factors vs. non CVD risk factors associated with various anthropometric measures of obesity by sex

Men	BMI<28.1	BMI≥28.1	WC<100.0	WC≥100.0	WHR<0.96	WHR≥0.96	WHTR<0.57	WHTR≥0.57
Hypertension	1.00 (ref)	2.75 (2.24, 3.37)***	1.00 (ref)	2.77 (2.25, 3.40)***	1.00 (ref)	2.40 (1.96, 2.93)***	1.00 (ref)	2.90 (2.36, 3.55)***
Dyslipidemia	1.00 (ref)	1.20 (0.94, 1.53)	1.00 (ref)	1.22 (0.96, 1.55)	1.00 (ref)	1.22 (0.96, 1.56)	1.00 (ref)	1.07 (0.84, 1.36)
Diabetes mellitus	1.00 (ref)	3.27 (2.30, 4.71)***	1.00 (ref)	2.78 (1.99, 3.92)***	1.00 (ref)	3.29 (2.29, 4.84)***	1.00 (ref)	3.96 (2.75, 5.81)***
≥1 risk factor	1.00 (ref)	2.66 (1.84, 3.93)***	1.00 (ref)	2.52 (1.72, 3.77)***	1.00 (ref)	2.36 (1.66, 3.41)***	1.00 (ref)	2.43 (1.69, 3.55)***
≥2 risk factors	1.00 (ref)	2.73 (2.23, 3.35)***	1.00 (ref)	2.68 (2.18, 3.29)***	1.00 (ref)	2.43 (1.98, 2.98)***	1.00 (ref)	2.74 (2.24, 3.37)***
Women	BMI<27.5	BMI≥27.5	WC<87.0 cm	WC≥87.0 cm	WHR<0.85	WHR≥0.85	WHTR<0.54	WHTR≥0.54
Hypertension	1.00 (ref)	3.19 (2.76, 3.70)***	1.00 (ref)	3.76 (3.25, 4.37)***	1.00 (ref)	2.58 (2.23, 2.98)***	1.00 (ref)	3.50 (3.01, 4.06)***
Dyslipidemia	1.00 (ref)	0.96 (0.80, 1.14)	1.00 (ref)	1.02 (0.85, 1.21)	1.00 (ref)	1.06 (0.89, 1.27)	1.00 (ref)	1.04 (0.87, 1.23)
Diabetes mellitus	1.00 (ref)	3.46 (2.49, 4.89)***	1.00 (ref)	3.76 (2.67, 5.38)***	1.00 (ref)	4.09 (2.91, 5.86)***	1.00 (ref)	3.48 (2.46, 5.03)***
≥1 risk factor	1.00 (ref)	1.79 (1.41, 2.29)***	1.00 (ref)	2.02 (1.59, 2.59)***	1.00 (ref)	1.82 (1.43, 2.34)***	1.00 (ref)	1.94 (1.53, 2.46)***
≥2 risk factors	1.00 (ref)	2.91 (2.51, 3.39)***	1.00 (ref)	3.44 (2.96, 4.01)***	1.00 (ref)	2.56 (2.20, 2.97)***	1.00 (ref)	3.24 (2.78, 3.79)***

Abbreviations: BMI, body mass index (kg/m^{-2}); WC, waist circumference (cm); WHR, waist-to-hip ratio; WHTR, waist-to-height ratio; * $P < 0.05$; ** $P < 0.01$; *** $P < 0.001$.

Figure S1 Data selection process in the main and the sensitivity analyses

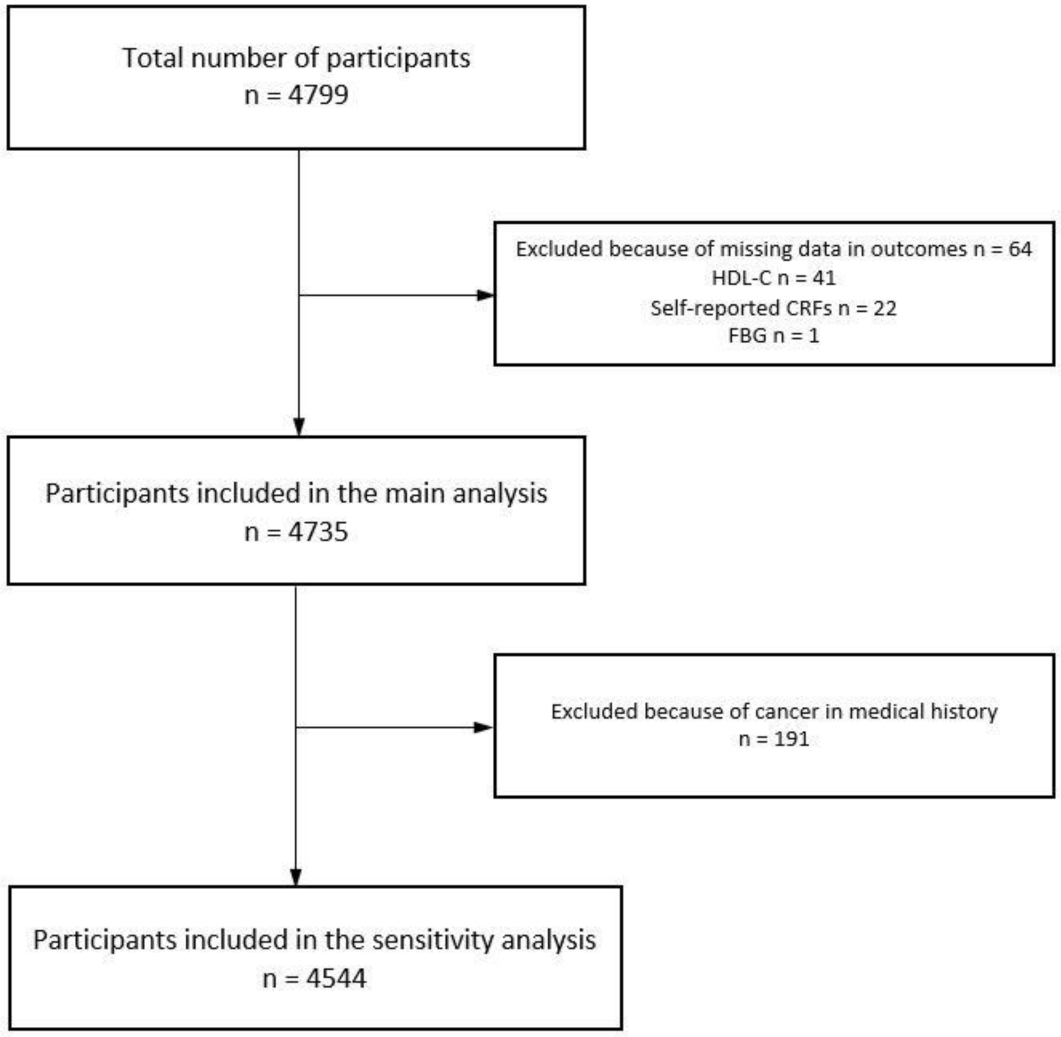

Table S5 Standard cut-off values for anthropometric measures of obesity, separately for men and women

Gender	BMI		WC		WHR		WHTR	
Men	<30.0	≥30.0	<94.0	≥94.0	<0.85	≥0.85	<0.50	≥0.50
Women	<30.0	≥30.0	<80.0	≥80.0	<0.75	≥0.75	<0.50	≥0.50

Abbreviations: BMI, body mass index (kg/m^{-2}); WC, waist circumference (cm); WHR, waist-to-hip ratio; WHTR, waist-to-height ratio.

Table S6 Methods of laboratory measurements

Parameter	Method
Fasting blood glucose (FBG)	Hexokinase
Total cholesterol (TC)	Cholesterol oxidase and cholesterol esterase
High density lipoprotein cholesterol (HDL-C)	Direct method with TOOS and surfactant
Triglycerides (TG)	Phosphoglyceride oxidase-peroxidase

Figure S2 Percentage body fat cut-off by at least one of CRF, separately for men and women

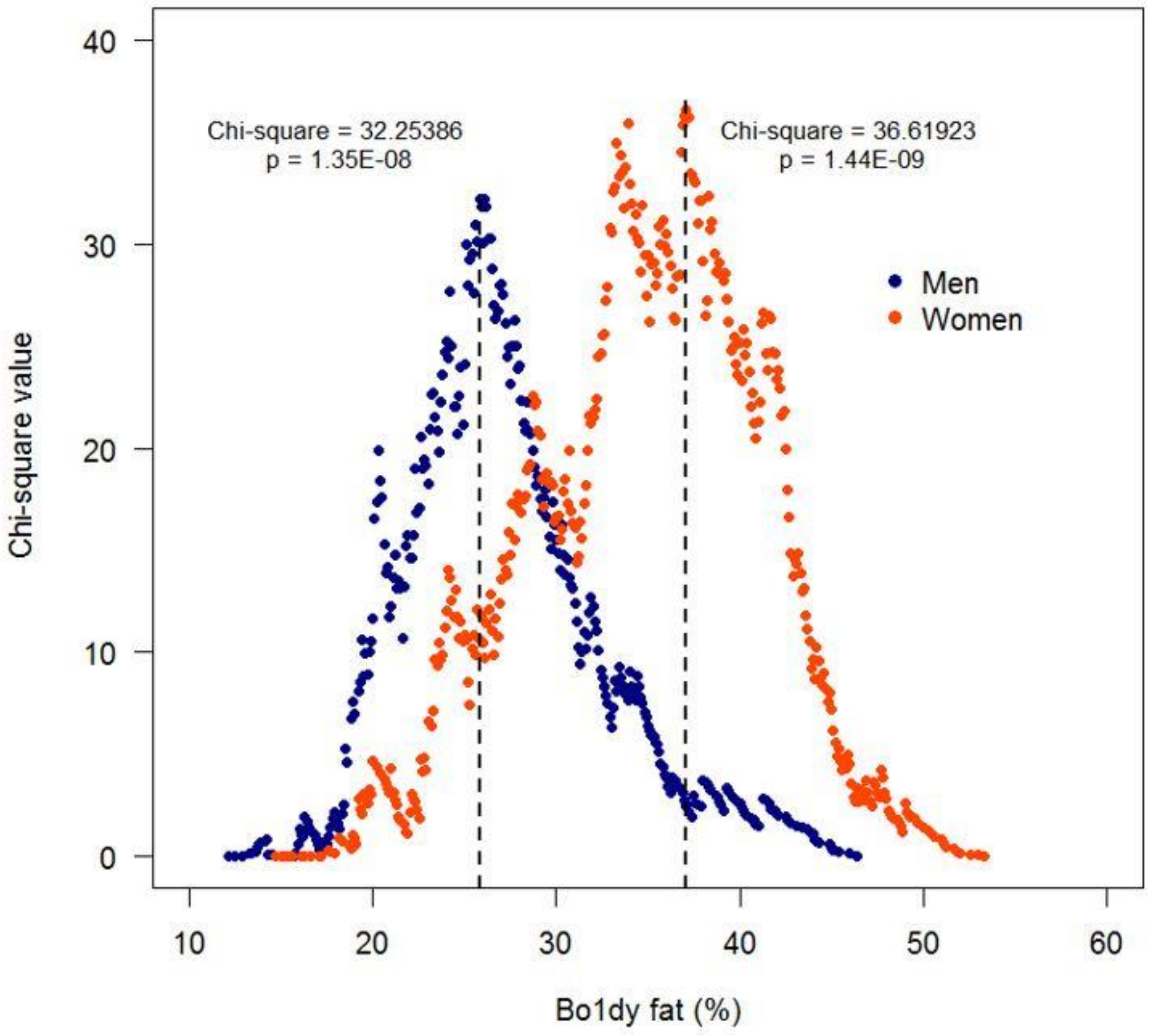

Figure S3 Percentage body fat cut-off by at least one of CRF based on the data after exclusion the participants with cancer in medical history, separately for men and women

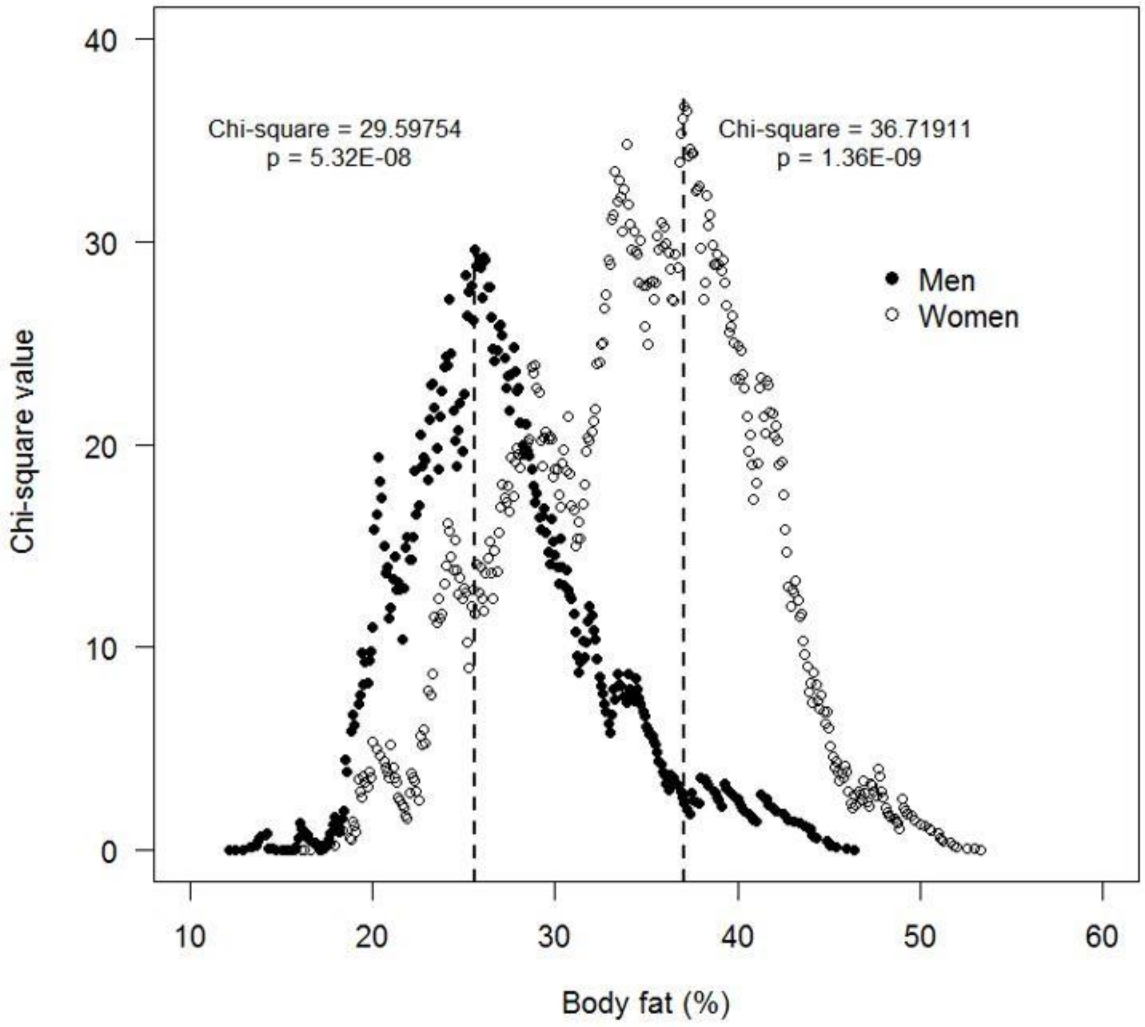

Note: All cases (n=191) with confirmed cancer in medical history were excluded from the study database. The sensitivity analysis was conducted based on 4544 of cases.

Table S7 Cut-off values (men only) for anthropometric measures of obesity based on optimal BF% cut-offs for screening CRFs after exclusion from the database all cases with cancer in medical history

Variable	AUC (95% CI)	Optimal cut-off	Sensitivity (%)	Specificity (%)	Youden (%)	DLR (+)	DLR (-)
BMI	0.913 (0.899, 0.927) [a]	28.1	80.3	87.0	67.3	6.2	0.2
WC	0.886 (0.870, 0.902) [a]	100.0	72.0	88.3	60.4	6.2	0.3
WHR	0.783 (0.760, 0.805) [a]	0.96	73.0	69.7	42.8	2.4	0.4
WHTR	0.892 (0.876, 0.908) [a]	0.57	77.9	84.2	62.1	4.9	0.3

Notes: There were 191 cases with confirmed cancer in medical history.
Abbreviations: BMI, body mass index (kg/m^{-2}); WC, waist circumference (cm), WHR, waist-to-hip ratio; WHTR, waist-to-height ratio; AUC, Area Under the Curve, DLR (+), positive diagnostic likelihood ratio; DLR (-), negative diagnostic likelihood ratio; [a] $P < 0.001$.
